# Comparison of 2 thromboelastography methods using patient and control samples

**DOI:** 10.1016/j.rpth.2025.102843

**Published:** 2025-03-30

**Authors:** Robert Frick, Brittany Washburn, Dennis Plocher, Jonathan K. Zoller, Jason Gillihan, Michael Dombrowski, Charles Eby, Christopher W. Farnsworth

**Affiliations:** 1 Department of Pathology & Immunology, Washington University School of Medicine, St. Louis, Missouri, USA; 2 Department of Laboratories, Barnes Jewish Hospital, St. Louis, Missouri, USA; 3 Department of Anesthesiology, Washington University in St. Louis, St. Louis, Missouri, USA; 4 Department of Obstetrics and Gynecology, Washington University in St. Louis, St. Louis, Missouri, USA

**Keywords:** blood product utilization, coagulation, method comparison, thromboelastometry, transfusion

## Abstract

**Background:**

Viscoelastic testing at point-of-care is associated with reduced blood loss and blood product transfusions. The ROTEM (Werfen) sigma is a cartridge-based system that may facilitate point-of-care use, but limited studies exist comparing the sigma with the predicated ROTEM delta.

**Objectives:**

We compared the performance of the ROTEM delta with that of the sigma.

**Methods:**

Citrated blood was collected from 20 healthy donors and patients during liver transplants (*n* = 17), obstetrics (*n* = 15), cardiovascular (*n* = 9), and trauma surgeries (*n* = 10). A method comparison was performed using the delta as the predicate. Imprecision was assessed at 2 levels for each assay. Manufacturer reference intervals were verified using 20 healthy donors. An algorithm used for cardiovascular surgery with the delta was compared with the sigma.

**Results:**

The coefficient of variation was <10% for all assays/parameters except for the thromboelastometry with extrinsic activation (EXTEM) clotting time (10.3%) and EXTEM amplitude (A)5 (10.2%). Reference intervals for the delta and sigma were comparable to manufacturer claims. The Pearson r comparing the delta and sigma exceeded .85 for all parameters/assays except for thromboelastometry with cytochalasin D-mediated platelet inhibition (FIBTEM) A10 (.77; 95% CI, .66-.86), FIBTEM A20 (.78; 95% CI, .65-.87), and thromboelastometry with heparinase clotting time (.77; 95% CI, .61-.87). No difference was observed in extrapolated thresholds from the delta-guided algorithm. However, extrapolated sigma A5 parameters for EXTEM were 5 mm lower, and for FIBTEM were 1 mm lower than delta A10 parameters.

**Conclusion:**

The ROTEM delta and sigma devices had comparable performance. A negative bias was observed in the FIBTEM assay with lower extrapolated clinical decision points for a delta-guided treatment algorithm for the FIBTEM and EXTEM A5.

## Essentials

• Limited studies exist comparing the ROTEM delta and sigma in multiple clinical locations.
• There was a high degree of correlation between results from the 2 devices and low imprecision.
• Implementation of the A5 parameter relative to the A10 used in many hospitals resulted in some bias.
• The delta and sigma perform similarly, but algorithms may require modification if switching.

## 1 Introduction

Viscoelastic hemostatic assays (VHAs) were first introduced in 1948 and have been commercially available since the 1960s [1,2]. In contrast to conventional coagulation testing performed in a central hospital laboratory, VHAs can be performed near patients on whole blood specimens, reducing time to results [2]. Further, VHAs provide a multifaceted evaluation of clotting, including time to initial clot formation, clot strength, and fibrinolysis [[1], [2], [3], [4]]. VHA testing has been increasingly used in the settings of liver transplantation, cardiac surgery, trauma, and obstetrical bleeding, where evidence supports reduced allogeneic transfusion when used in conjunction with patient blood management algorithms to guide hemostatic resuscitation [1,2,[5], [6], [7], [8]]. Several studies have also demonstrated a reduction in blood loss and transfusions with VHAs when applied at the point-of-care (POC) compared with laboratory-based conventional coagulation testing, implying a potentially important role of VHA testing for reducing turnaround time when used in conjunction with a transfusion algorithm [6,9,10].

Recently, the Food and Drug Administration (FDA) cleared cartridge-based VHAs, such as the ROTEM sigma, for specific clinical indications. Predicate devices require manual pipetting and are often performed in clinical laboratories due to concerns of increased imprecision when performed by nonlaboratorians [11]. The use of a cartridge-based technology may improve the ease of use and reproducibility of test results. However, many published patient blood management algorithms are based on the ROTEM delta instrument, and studies specifically comparing the performance of the ROTEM delta and sigma devices are limited. Importantly, 3 studies noted significant biases in certain parameters that could impact transfusion practices if delta-based algorithms were not modified [[12], [13], [14]]. However, published studies comparing the devices are relatively few, with limited scope of patient cohorts, such as obstetrics or trauma only. Further, these previously published studies also pooled ROTEM assays, limiting interpretability. Finally, in some areas of the world, such as the United States, clearance of the ROTEM delta did not include some parameters, such as the amplitude (A) at 5 minutes (A5), which are now included as part of the sigma assay. For hospitals that are not able to report the A5 on the delta instrument, it is not clear how switching to the ROTEM sigma and the A5 parameter should be validated or operationalized. Importantly, establishing analytical equivalency between devices across all ROTEM assays from multiple patient cohorts is critical when transitioning from the delta to the sigma in hospital systems where VHA testing is used for multiple clinical indications.

Herein, we compared the ROTEM delta and sigma instruments using healthy controls and patients from liver transplant, obstetrics, cardiovascular, and trauma operating room settings. Further, we assessed the imprecision and verified the manufacturer reference intervals (RIs). Finally, the impact of switching from the ROTEM delta to sigma was assessed using a previously published ROTEM-guided transfusion algorithm and the results of the method comparison study.

## 2 Methods

### 2.1 Patient population

This study was performed as part of routine clinical verification/validation of a new assay, was not funded or designed by the manufacturer, and was deemed nonhuman subjects research by the Washington University Institutional Review Board (IRB). Healthy volunteers were prospectively collected for verification of manufacturer RIs, with a target of 20 volunteers to capture the 95th percentiles in accordance with Clinical and Laboratory Standards Institute document EP28-A3c [15]. Exclusion criteria included known disorders of hemostasis, anticoagulant use, or use of anticoagulant therapy within 48 hours. Patients undergoing surgery were included at the discretion of the attending anesthesiologist. Whole blood specimens were collected in 3 mL vacutainer tubes (BD) containing 3.2% sodium citrate by peripheral venipuncture (healthy volunteers) or from a previously sited radial arterial catheter line (patients). For cardiac surgical patients, samples were obtained at differing time points during surgery, including the pre- and postcardiopulmonary bypass periods. For patients undergoing liver transplantation, samples were obtained prior to graft reperfusion. All labor and delivery (L&D) patients had specimens procured during cesarean section. For the method comparison study, 2 tubes of blood were collected. Patient demographics were collected from the electronic medical record by a blinded observer. Due to IRB constraints, medical record information was not available for the healthy volunteers. We targeted recruitment of 10 subjects per clinical location, with a goal of at least 40 specimens to identify relevant bias in accordance with Clinical and Laboratory Standards Institute EP09C ED3 [16].

### 2.2 Study design

To reflect clinical use, studies were performed in simulated “real-world” conditions. Six ROTEM sigma devices were assessed during this study and compared with 3 ROTEM delta devices. The delta and sigma devices were tested according to the manufacturer’s instructions. For the ROTEM delta, the thromboelastometry with heparinase (HEPTEM) lyophilized reagent was reconstituted and kept at 4 °C until testing. The remainder of reagents were purchased as liquid-based reagents. Devices were chosen at random, and all testing was performed by technologists integrated into their routine workflow. For clinical samples, the delta instrument was tested first, and the sigma instrument was tested within 15 minutes. Healthy control samples were analyzed simultaneously on both instruments. A minimum of 5 patient specimens were tested on each sigma instrument.

### 2.3 ROTEM parameters

The following assays were tested on both the ROTEM delta and sigma: the thromboelastometry with extrinsic activation (EXTEM) assay activates the extrinsic pathway via tissue factor; the thromboelastometry with cytochalasin D-mediated platelet inhibition (FIBTEM) assay is similar to the EXTEM assay, but it excludes the effects of platelets using the inhibitor cytochalasin D; the thromboelastometry with intrinsic activation (INTEM) assay activates the intrinsic pathway via ellagic acid; and the HEPTEM assay is the same as the INTEM assay, but it activates the intrinsic pathway after neutralization of heparin by heparinase in the presence of ellagic acid. The following parameters were tested for each assay, as indicated: the clotting time (CT) provides the time until the initiation of clotting, and the A indicates the clot firmness at the indicated time in minutes. The delta is FDA-cleared to report the A at 10 minutes (A10) and 20 minutes (A20), while the sigma is cleared to report the A5, A10, and A20. As a result, the A10 parameter on the delta was compared with the A10 and A5 on the sigma. The maximum clot firmness (MCF) is the maximum A. The lysis at 60 minutes and maximum lysis indicate clot stability and fibrinolysis, respectively.

### 2.4 Imprecision studies

ROTROL quality control (QC) material for precision studies was integrated into the training of technologists. QC materials for normal and abnormal levels were reconstituted per manufacturer instructions and analyzed on 6 sigma instruments for 1 run per day per control level across 5 days and on 1 instrument 5 times per control level on 1 day. An initial target coefficient of variation (CV) of 10% was used for sigma imprecision studies based on manufacturer claims and the previous performance of the delta instrument.

### 2.5 ROTEM-guided cardiovascular operating room transfusion guidelines

The impact of switching to the ROTEM sigma from the delta was assessed using extrapolation from the Deming regression best-fit lines from method comparisons. Clinical decision limits used were based on a previously published algorithm for cardiovascular operating room (CVOR) patients [7]. An ideal threshold for the A5 parameter on the ROTEM sigma was assessed from Deming regression of the A5 relative to the A10 results of the ROTEM delta instrument in order to compare the earliest reported parameters on each device. The ROTEM delta A5 is not cleared in the US, and thus, results for this parameter were not available to the investigators.

### 2.6 Statistical analysis

For (RI) verification, identification of outliers involved a multivariate approach to capture both abnormal results and abnormal correlations between results. To achieve this, a Mahalanobis distance was calculated for each patient result within each assay [17]. These distances were plotted, and outliers were removed by manual inspection. Volunteer specimens with outliers in 1 or more assays were reviewed for potential preanalytic errors, and participating volunteers were invited for additional interviews to evaluate the clinical context of the results. Samples were selected for exclusion after a review of the results and interview findings. For precision studies, within-day CV and within-laboratory CV (ie, between days and between instruments, respectively) were calculated on the sigma instrument. RIs were compared between healthy donors and the manufacturer’s claims for each device. The published RIs for each device can be found in Table 1. For method comparison studies, data were analyzed using Pearson r correlation, Deming regression, and Bland–Altman plots to evaluate bias. If a result for an assay exceeded the analytic measuring range, it was excluded from method comparison analysis. Similarly, if there was an error in analysis for the sigma or delta that specific analyte was excluded for that patient. The number of specimens included for each assay comparison is indicated. A best-fit Deming regression line was used to extrapolate clinical decision points from the previously identified CVOR transfusion guidelines [7] from the delta parameters to their sigma equivalents. Statistics were performed using GraphPad Prism v10.

**Table 1:** ROTEM sigma imprecision.

Assay	Parameter	ROTROL (normal)				ROTROL (abnormal)			
		N	%CV (within-run)	N	%CV (within-lab)^a^	N	%CV (within-run)	N	%CV (within-lab)^a^
EXTEM	CT (s)	5	8.5	45	10.3	5	2.2	40	3.6
	A5 (mm)	5	2.8	45	3.5	5	10.2	40	7.0
	A10 (mm)	5	1.4	45	2.9	5	8.8	40	6.2
	A20 (mm)	5	1.5	45	2.9	5	8.3	42	5.9
	MCF (mm)	5	5.5	40	4.8	5	5.8	40	7.1
INTEM	CT (s)	5	3.5	45	4.2	5	2.1	40	2.5
	A5 (mm)	5	2.3	45	3.6	5	3.1	40	4.7
	A10 (mm)	5	2.3	45	3.6	5	3.2	40	4.2
	A20 (mm)	5	2.1	45	3.0	5	3.1	42	3.9
	MCF (mm)	5	5.7	40	5.0	5	1.8	40	6.1
FIBTEM	A5 (mm)	5	3.8	45	6.7	5	6.6	40	5.9
	A10 (mm)	5	2.6	45	6.0	5	4.2	40	5.4
	A20 (mm)	5	3.0	45	5.7	5	5.0	42	4.9
	MCF (mm)	5	3.3	40	5.9	5	5.6	40	6.2
HEPTEM	CT (s)	5	4.4	45	4.1	5	1.9	40	2.6
	A5 (mm)	5	3.4	45	2.9	5	3.7	40	3.9
	A10 (mm)	5	3.0	45	2.9	5	3.2	40	3.3
	A20 (mm)	5	2.8	45	2.9	5	1.9	42	3.3
	MCF (mm)	5	2.4	40	5.0	5	3.8	40	5.4

A5, amplitude at 5 minutes; A10, amplitude at 10 minutes; A20, amplitude at 20 minutes; CT, clotting time in seconds; CV, coefficient of variation; EXTEM, thromboelastometry with extrinsic activation; FIBTEM, thromboelastometry with cytochalasin D-mediated platelet inhibition; HEPTEM, thromboelastometry with heparinase; INTEM, thromboelastometry with intrinsic activation; Lab, laboratory; MCF, maximum clot formation; ROTROL, ROTEM manufacturer quality control materials, normal and abnormal.

a Within-lab CV is defined as the imprecision across all 6 instruments, with a minimum of 5 runs per instrument across a minimum of 5 days.

## 3 Results

### 3.1 Imprecision

Results from the precision study data are summarized in Table 1. None of the results for each assay, parameter, or instrument had a CV that exceeded 10%, except for the EXTEM CT with normal control (10.3% for within-laboratory) and the EXTEM A5 with abnormal control (10.2% for within-run). The imprecision for each instrument can be found in Supplementary Table S1.

### 3.2 RI verification

A total of 23 specimens were collected for RI verification. Of these, 3 specimens were determined to be outliers, and 3 were excluded from analysis. Calculated Mahalanobis distances are presented in Supplementary Table S2. A summary of the RI data is presented in Table 2. The most out-of-range values relative to the manufacturer RI claims for each assay were observed for the INTEM CT (*n* = 4) and HEPTEM CT (*n* = 3) on the ROTEM sigma. However, the same specimens were outside of the manufacturer-defined RIs for the ROTEM delta. Experimentally derived RIs for the delta and sigma were comparable to the manufacturer’s claims.

**Table 2:** Reference intervals from healthy volunteers.

Assay	Parameter	Manufacturer delta	Experimental delta (*N*= 20)	Manufacturer sigma	Experimental sigma (*N*= 20)
EXTEM	CT (s)	43-82	55-86	51-73	57-102
	A5 (mm)	NA	NA	33-52	33-54
	A10 (mm)	NA	43-68	45-62	45-64
	A20 (mm)	50-70	51-73^a^	54-69	53-71
	MCF (mm)	52-70	54-74^a^	57-72	56-73
	LI60 (%)	NA	89-98	94-100	93-100
	ML (%)	NA	2-11	0-6	0-7
INTEM	CT (s)	122-208	171-229^a^	139-205	173-221^a^
	A5 (mm)	NA	NA	36-54	36-54
	A10 (mm)	NA	44-66	46-63	46-65
	A20 (mm)	51-72	52-71	53-68	52-70
	MCF (mm)	51-72	52-71	55-70	54-71
	LI60 (%)	NA	89-99	93-100	92-99
	ML (%)	NA	1-11	0-7	2-9
FIBTEM	A5 (mm)	NA	NA	5-16	7-16
	A10 (mm)	NA	10-24	6-17	7-15
	A20 (mm)	7-24	11-25	6-18	8-17
	MCF (mm)	7-24	10-25	6-19	8-17
HEPTEM	CT (s)	NA	186-244^a^	141-215	170-229^a^
	A5 (mm)	NA	NA	33-51	35-53
	A10 (mm)	NA	42-63	44-61	44-63
	A20 (mm)	NA	48-66	52-67	52-69
	MCF (mm)	NA	48-67	54-69	54-69

A5, amplitude at 5 minutes; A10, amplitude at 10 minutes; A20, amplitude at 20 minutes; CT, clotting time in seconds; EXTEM, thromboelastometry with extrinsic activation; FIBTEM, thromboelastometry with cytochalasin D-mediated platelet inhibition; HEPTEM, thromboelastometry with heparinase; INTEM, thromboelastometry with intrinsic activation; LI60, lysis at 60 minutes; MCF, maximum clot formation; ML, maximum lysis; NA, not available.

a ≥2 values out of range compared with the manufacturer reference intervals.

### 3.3 Method comparison study

**Figure 1:**
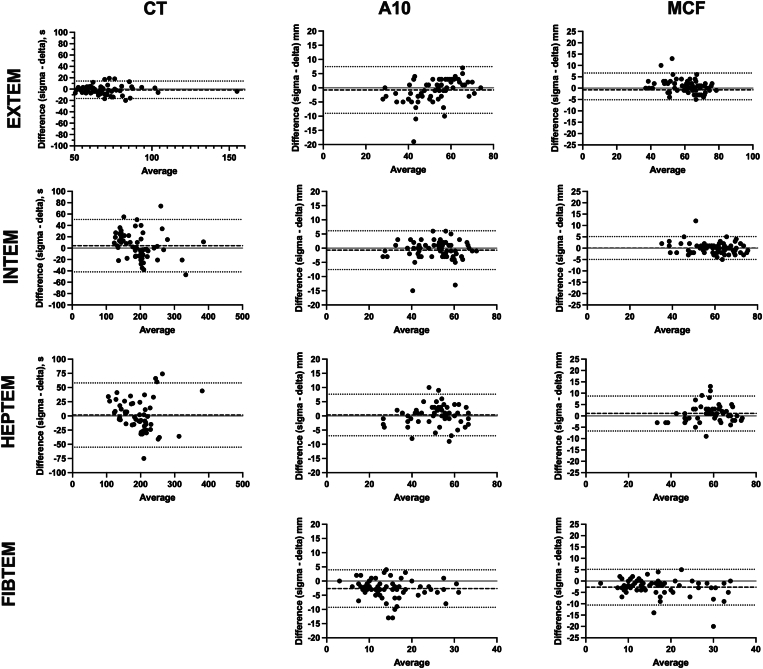
Bland–Altman comparisons between the ROTEM delta and sigma. Seventy-one patients were assessed on each instrument from multiple clinical locations. Healthy controls, *n* = 20; Labor and Delivery (L&D), *n* = 15; cardiovascular operating room, *n* = 9; liver transplant, *n* = 17; and trauma, *n* = 10. The black dashed line is the observed bias, the dotted black line is the 95% CI, and the gray line is at 0. A10, amplitude at 10 minutes; CT, clotting time in seconds; EXTEM, thromboelastometry with extrinsic activation; FIBTEM, thromboelastometry with cytochalasin D-mediated platelet inhibition; HEPTEM, thromboelastometry with heparinase; INTEM, thromboelastometry with intrinsic activation; MCF, maximum clot formation.

There were 20 samples from healthy volunteers and an additional 51 patients recruited from clinical floors; 15 were from labor and delivery, 9 from the CVOR, 17 from liver transplant, and 10 from trauma. The median age was 49 years (IQR, 34-70), 49% were male, and 88% self-identified as White, with the remaining identifying as Black. The median international normalised ratio was 1.5 (1.2-1.83), the median partial thromboplastin time was 34 seconds (28-37.5), the median fibrinogen was 193 mg/dL (160-272), and the median platelet count was 113 k/cumm (78-208; Supplementary Table S3). The youngest cohort of patients was from L&D, with a median age of 34 years, and the oldest were CVOR patients, with a median age of 70 years. Laboratory results were comparable between groups with the exception of platelets, which were ∼2× higher in L&D patients relative to other groups. The method comparison data are summarized in Table 3. The Pearson r exceeded .85 for all parameters on all assays, with exception of the HEPTEM CT (.847; 95% CI, .75-.908). Bias for all parameters in each assay was <3 Units (seconds or mm) and the CIs for the bias crossed 0 for each parameter in each assay (Figure 1). Deming regression slopes and intercepts can be found in Supplementary Table S4 and Supplementary Figure. Stratification of the method comparison results by indication (healthy controls, obstetrics, CVOR, liver transplant, and trauma) demonstrated no difference in results for the assessed parameters. However, for the healthy controls, a subset of patients had higher results on the delta relative to the sigma in the FIBTEM assay for A10 and MCF.

**Table 3:** ROTEM sigma vs delta method comparison summary statistics.

Assay	Parameter	Samples	Pearson r	95% CI	R^2^	Absolute bias	95% CI of bias
EXTEM	CT (s)	69	.893	.83-.93	.797	−1.22	−16.6 to 14.17
	A10 (mm)	69	.937	.89-.96	.878	−0.77	−8.99 to 7.45
	A20 (mm)	67	.958	.93-.97	.918	0.7	−6.82 to 8.22
	MCF (mm)	69	.957	.93-.97	.915	0.76	−5.14 to 6.66
INTEM	CT (s)	61	.908	.85-.94	.823	4.36	−42.17 to 50.89
	A10 (mm)	65	.944	.91-.966	.892	−0.71	−7.53 to 6.12
	A20 (mm)	64	.968	.948-.98	.937	−0.7	−6.6 to 5.19
	MCF (mm)	66	.966	.945-.979	.934	0.06	−4.97 to 5.09
FIBTEM	A10 (mm)	54	.864	.788-.915	.748	−2.66	−9.26 to 3.95
	A20 (mm)	54	.858	.779-.91	.736	−2.82	−10.48 to 4.83
	MCF (mm)	68	.869	.795-.917	.755	−2.71	−10.58 to 5.16
HEPTEM	CT (s)	55	.847	.75-.908	.593	−1.47	−55.12 to 58.07
	A10 (mm)	55	.936	.89-.96	.876	0.31	−7.02 to 7.64
	A20 (mm)	55	.908	.84-.948	.824	1.15	−5.74 to 8.03
	MCF (mm)	56	.915	.859-.95	.837	1.08	−6.56 to 8.73

A total of 71 specimens were analyzed. For some measurements, specimens exceeded the analytic measuring range and were excluded. Some specimens were excluded because the paired result on the methods was not available due to an error or a technologist ending the run prior to generation of the result.

A10, amplitude at 10 minutes; A20, amplitude at 20 minutes; CT, clotting time in seconds; EXTEM, thromboelastometry with extrinsic activation; FIBTEM, thromboelastometry with cytochalasin D-mediated platelet inhibition; HEPTEM, thromboelastometry with heparinase; INTEM, thromboelastometry with intrinsic activation; MCF, maximum clot formation.

**Figure 2:**
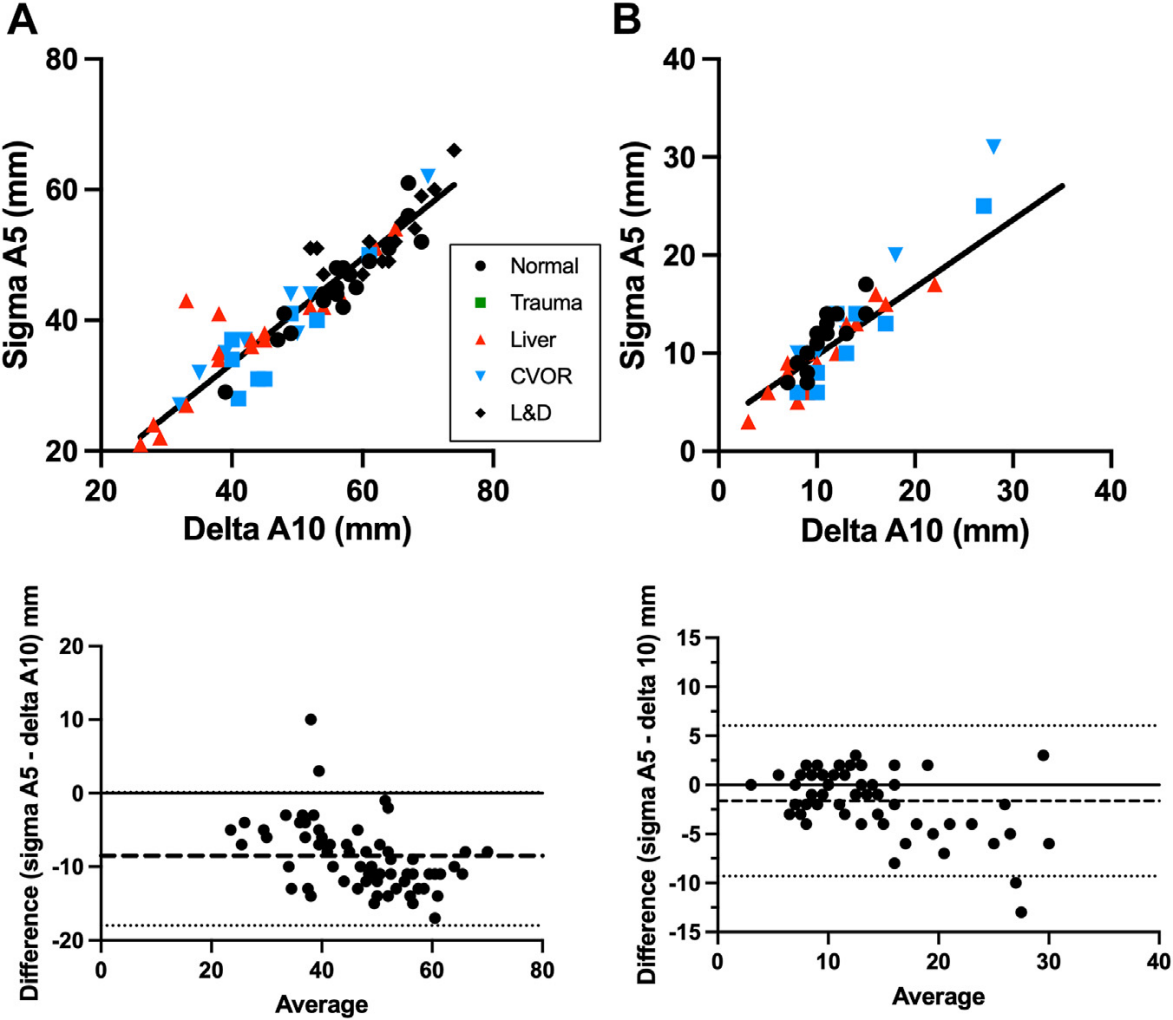
Comparison between sigma A5 and delta A10. Samples were run on the delta and sigma instruments for (A) EXTEM and (B) FIBTEM. Shown are the Deming regression (top) and Bland–Altman plots (bottom) with the bias (dashed line) and 95% CI (dotted lines).A5, amplitude at 5 minutes; A10, amplitude at 10 minutes; CVOR, cardiovascular operating room; EXTEM, thromboelastometry with extrinsic activation; FIBTEM, thromboelastometry with cytochalasin D-mediated platelet inhibition.

A comparison of EXTEM A5 on the sigma instrument with EXTEM A10 on the delta instrument revealed a slope of 0.8 (0.7-0.89), an intercept of 1.22 (−3.31 to 5.75), and an average bias of −8.92 (−17.99 to 0.14; Figure 2A). A comparison of FIBTEM A5 on the sigma instrument with FIBTEM A10 on the delta instruments revealed a slope of 1.02 (0.83-1.22), an intercept of −0.36 (−2.52 to 1.8), and an average bias of −1.64 (−9.31 to 6.03; Figure 2B).

### 3.4 ROTEM-guided CVOR transfusion guidelines

Table 4 highlights the extrapolated values for the clinical decision points for the ROTEM sigma based on Deming regression relative to the ROTEM delta results. At a clinical decision point of 240 seconds for the INTEM CT, the extrapolated sigma value was 239 seconds (95% CI, 229-249 seconds). For the HEPTEM CT, a delta threshold of 240 seconds correlated with a threshold of 239 seconds (222-257 seconds) on the sigma, and for EXTEM CT, the threshold of 90 seconds correlated with a sigma threshold of 87 seconds (84-91 seconds). For the A10 parameter on the delta, the EXTEM assay threshold of 40 mm correlated with a sigma result of 43 mm (41-46 mm). Relative to the EXTEM A10 threshold of 40 mm, the sigma A5 extrapolated value was 35 mm (33-37 mm). In the delta FIBTEM assay, 8 mm correlated with a sigma threshold of 8 mm (6-10 mm). The FIBTEM A10 threshold of 8 mm on the ROTEM delta correlated with an A5 of 7 mm (5-9 mm) on the sigma.

**Table 4:** Conversions of common clinical decision-making thresholds from ROTEM delta thromboelastometry with cytochalasin D-mediated platelet inhibition to ROTEM sigma equivalent parameters.

Parameter	Delta threshold	Extrapolated sigma (95% CI)
INTEM CT	240	239 (229-249)
HEPTEM CT	240	239 (222-257)
EXTEM CT	90	87 (84-91)
EXTEM A10	40	43 (41-46)
EXTEM A10 to sigma A5	40	35 (33-37)
FIBTEM A10	8	8 (6-10)
FIBTEM A10 to sigma A5	8	7 (5-9)

A5, amplitude at 5 minutes; A10, amplitude at 10 minutes; CT, clotting time in seconds; EXTEM, thromboelastometry with extrinsic activation; FIBTEM, thromboelastometry with cytochalasin D-mediated platelet inhibition; HEPTEM, thromboelastometry with heparinase; INTEM, thromboelastometry with intrinsic activation.

## 4 Discussion

The transition to cartridge-based VHA testing on the ROTEM sigma offers several potential advantages over the ROTEM delta. However, for laboratories transitioning from the predicate delta device, there are challenges, including a paucity of publications comparing the performance of the sigma to the delta. Further, VHA-guided transfusion algorithms that were developed on the delta platform must be adapted for any changes in assay performance when switching to the sigma. In this study, we found comparable performance between the ROTEM delta and sigma devices, with the exception of a bias in the FIBTEM parameters and the HEPTEM CTs. An important strength of this study was that it was a relatively large method comparison across multiple clinical settings: healthy controls, CVOR, obstetrics, trauma, and liver transplant patients. Importantly, the observed differences between the sigma and delta results, particularly the sigma EXTEM and FIBTEM A5 vs delta EXTEM and FIBTEM A10, imply that transfusion algorithms may require modification when switching instrumentation.

While the assays were largely comparable, we did observe negative bias in the FIBTEM assay on the sigma relative to the delta. Parameters on the ROTEM sigma were 13% to 15% lower than the delta predicate device. This finding is mirrored in the changes between the sigma and delta manufacturer reference ranges for the FIBTEM (delta A10: 7-23 > sigma A10: 6-17). Interestingly, this effect was significantly larger in healthy controls (−26% to −30%) than clinical patient samples (−7% to −8%). The source of this difference is unknown and must be verified by other studies. Nonetheless, similar findings were reported in other studies. Gillissen et al. [13] reported a negative bias of −3 mm for the sigma FIBTEM A10, closely resembling −2.4 mm negative bias observed here. Similarly, a study by Modica et al. [12] reported a −10% bias for the FIBTEM parameters on the sigma relative to the delta. In contrast, a subsequent study by Auty et al. [14], examining multiple fibrinogen assays in trauma patients, found a positive bias in the sigma compared with the delta that increased over time (from −0.75 mm up to −3.57 mm at the 10th timepoint).

Taken together, the results from this study verify others’ findings, particularly for the FIBTEM assay that differences in results exist between the ROTEM delta and sigma instruments that may depend on the patient’s clinical setting.

The differences observed between assays on the sigma and delta imply a potential need to alter thresholds for clinical decision limits for transfusion protocols. To this end, we extrapolated values from the best-fit Deming regression for patient samples of the delta and sigma from previously published algorithms [7]. Using this approach, we observed no change in thresholds on the sigma with the exception of the EXTEM A5 by 5 mm and the FIBTEM A5 by 1 mm. This is comparable with a previous study which found that transition to the sigma with no change from the delta-based algorithm would potentially increase transfusion of cryoprecipitate by 10% [18]. Taken together, these data imply a need for institutions switching to the sigma device from previous ROTEM models to consider modifying clinical cutoffs and decision limits [11,[19], [20], [21], [22], [23], [24], [25], [26]].

There are several potential advantages to cartridge-based iterations of viscoelastic assays. An important finding from this study was that there was an overall low imprecision of <10% across all 6 instruments and more than 20 trained users. This contrasts with prior studies using the ROTEM delta which showed considerable interobserver imprecision exceeding 8% to 11% for some parameters [11,27,28]. This may imply that the ROTEM sigma is viable for use at the POC, which could facilitate more rapid turnaround times. It is worth noting that the lower imprecision with the sigma was achieved during a training period using untrained users; thus, the imprecision may be lower with routine use than what is presented in this study. However, further studies are needed to demonstrate the efficacy of this approach. An additional consideration for turnaround time is that the ROTEM sigma reports an A5, whereas the delta only reports an A10. Herein, we demonstrate comparable results between the A5 and A10 on the sigma instrument, with a near-perfect slope of 1.0 and an intercept of 0. Interestingly, even a comparison between the A5 on the sigma relative to the A10 on the delta yielded comparable, albeit somewhat diminished, results for both the FIBTEM and EXTEM. Using the extrapolation approach, we were also able to identify an A of 7 mm for the FIBTEM A5 and 35 mm for the EXTEM A5. The strong intercorrelation between the A5, A10, and MCF parameters have been previously noted [29]. Together, this implies that the A5 parameters may be implemented in clinical decision-making algorithms in place of A10 parameters, further reducing time until actionable results. However, it is also worth noting potential drawbacks of switching to the sigma; namely, there may be a potential increase in cost per test, which is likely institution-specific. Further, at this time, there is no anti-fibronolysis assay on the sigma, an assay commonly used in conjunction with the EXTEM assay on the delta instrument to assess fibrinolysis. Finally, the sigma instrument has a greater volume requirement (full-tube of citrated blood) than the delta (∼300 μL of blood), which may have limitations in some settings, such as pediatrics.

In this study, we verified the ROTEM sigma’s manufacturer RIs and found that they closely matched a healthy cohort of 20 participants. It is worth noting that there were 4 healthy donors with elevated INTEM and HEPTEM CT parameters, exceeding the target of 10% or fewer values outside of the manufacturer’s RI. As both INTEM and HEPTEM evaluate the intrinsic pathway, with HEPTEM containing heparinase to eliminate the effect of heparin, concurrent elevation in parameters in both assays for nonheparinized donors is an expected finding. Interestingly, the ROTEM delta results had similar elevations for the same assays and parameters in these donors. Alternative explanations for these results include contaminated specimens or analytic errors. Nonetheless, the observed out-of-range values (sigma INTEM CT 210-221 seconds) fall below common thresholds identified in the literature for intervention (INTEM CT > 240 seconds), which is often also compared as a ratio relative to the HEPTEM CT. The reason for these differences is unclear but may be due to the use of lyophilized reagent in the sigma as opposed to liquid reagents in the delta. Together, these results imply that the manufacturer-suggested RIs are sufficient for the patient population assessed in this study but should still be assessed locally by each laboratory adopting the ROTEM sigma device.

There were several limitations associated with this study. This was a single-center study with all associated limitations, including patient demographics specific to 1 institution and limited number of operators. Further, the majority of the testing was performed by trained technologists in the laboratory as opposed to the intended operators at the POC. As a result, precision and accuracy of clinical staff during their normal workflow may be reduced relative to those in this study. We also only used and verified thresholds from a single published algorithm from CVOR. Finally, due to limitations in study design and IRB requirements, we were not able to assess results from this study with outcomes such as bleeding, mortality, or need for transfusion. Thus, the test that better identifies and correlates with outcomes could not be determined from this study. Finally, due to lack of FDA approval of the A5 delta parameter, a direct comparison with the sigma A5 could not be made. Thus, the approach for establishing relevant A5-based sigma cutoffs here may be useful for other US-based hospitals, but it has numerous limitations from a global perspective where the A5 is reportable on the delta. Studies that directly compare the delta A5 and sigma A5 would be of greater value and are needed.

## 5 Conclusion

The ROTEM delta and sigma instruments demonstrate comparable analytic performance across multiple clinical settings with modest variation in some assays/parameters. For parameters demonstrating differences, algorithms may need to be adjusted, or alternatively, regression analysis may be useful for distinguishing the ideal clinical cutoffs on the sigma instrument.
