# Supplementary material for: Comparison of 2 thromboelastography methods using patient and control samples
Source: Res Pract Thromb Haemost. 2025 Mar 30;9(3):102843. doi: 10.1016/j.rpth.2025.102843 (PMC12056965; doi:10.1016/j.rpth.2025.102843)
Supplement: Supplementary Material [file mmc1.docx]

**Supplemental Material**

**Supplemental Table 1: ROTEM Sigma Precision Study Summary Data, By Instrument**

|  |  |  | ROTROL Normal | | ROTROL Abnormal | |
| --- | --- | --- | --- | --- | --- | --- |
| Instrument | **Channel** | **Parameter** | **Samples** | **%CV** | **Samples** | **%CV** |
| 11650 | EXTEM | CT | 5 | 4.47 | 5 | 3.35 |
|  |  | A5 | 5 | 2.36 | 5 | 5.28 |
|  |  | A10 | 5 | 2.21 | 5 | 3.46 |
|  |  | A20 | 5 | 2.02 | 5 | 4.54 |
|  |  | MCF | 5 | X^1^ | 5 | X |
|  | INTEM | CT | 5 | 3.40 | 5 | 1.99 |
|  |  | A5 | 5 | 2.38 | 5 | 2.54 |
|  |  | A10 | 5 | 2.39 | 5 | 3.37 |
|  |  | A20 | 5 | 2.08 | 5 | 2.62 |
|  |  | MCF | 5 | X | 5 | X |
|  | FIBTEM | A5 | 5 | 3.01 | 5 | 6.43 |
|  |  | A10 | 5 | 1.79 | 5 | 5.70 |
|  |  | A20 | 5 | 2.52 | 5 | 5.15 |
|  |  | MCF | 5 | X | 5 | X |
|  | HEPTEM | CT | 5 | 3.54 | 5 | 2.68 |
|  |  | A5 | 5 | 2.74 | 5 | 3.96 |
|  |  | A10 | 5 | 2.02 | 5 | 3.24 |
|  |  | A20 | 5 | 1.86 | 5 | 2.53 |
|  |  | MCF | 5 | X | 5 | X |
|  |  |  |  |  |  |  |
| 11651 | EXTEM | CT | 5 | 5.96 | 5 | 1.80 |
|  |  | A5 | 5 | 2.28 | 5 | 3.52 |
|  |  | A10 | 5 | 1.63 | 5 | 3.12 |
|  |  | A20 | 5 | 1.50 | 5 | 1.85 |
|  |  | MCF | 5 | X | 5 | X |
|  | INTEM | CT | 5 | 3.25 | 5 | 1.93 |
|  |  | A5 | 5 | 4.17 | 5 | 2.32 |
|  |  | A10 | 5 | 4.23 | 5 | 2.06 |
|  |  | A20 | 5 | 2.62 | 5 | 0.00 |
|  |  | MCF | 5 | X | 5 | X |
|  | FIBTEM | A5 | 5 | 3.02 | 5 | 3.37 |
|  |  | A10 | 5 | 2.74 | 5 | 4.13 |
|  |  | A20 | 5 | 2.38 | 5 | 2.94 |
|  |  | MCF | 5 | X | 5 | X |
|  | HEPTEM | CT | 5 | 3.67 | 5 | 2.26 |
|  |  | A5 | 5 | 1.69 | 5 | 1.93 |
|  |  | A10 | 5 | 1.58 | 5 | 1.71 |
|  |  | A20 | 5 | 1.46 | 5 | 3.13 |
|  |  | MCF | 5 | X | 5 | X |
|  |  |  |  |  |  |  |
| 11654 | EXTEM | CT | 5 | 8.57 | 5 | 2.22 |
|  |  | A5 | 5 | 2.77 | 5 | 10.19 |
|  |  | A10 | 5 | 1.36 | 5 | 8.84 |
|  |  | A20 | 5 | 1.55 | 5 | 9.11 |
|  |  | MCF | 5 | X | 5 | X |
|  | INTEM | CT | 5 | 3.55 | 5 | 2.07 |
|  |  | A5 | 5 | 2.27 | 5 | 3.07 |
|  |  | A10 | 5 | 2.26 | 5 | 3.19 |
|  |  | A20 | 5 | 2.11 | 5 | 3.13 |
|  |  | MCF | 5 | X | 5 | X |
|  | FIBTEM | A5 | 5 | 3.83 | 5 | 6.59 |
|  |  | A10 | 5 | 2.60 | 5 | 4.16 |
|  |  | A20 | 5 | 2.94 | 5 | 4.98 |
|  |  | MCF | 5 | X | 5 | X |
|  | HEPTEM | CT | 5 | 4.44 | 5 | 1.86 |
|  |  | A5 | 5 | 3.41 | 5 | 3.67 |
|  |  | A10 | 5 | 3.03 | 5 | 3.24 |
|  |  | A20 | 5 | 2.79 | 5 | 1.92 |
|  |  | MCF | 5 | X | 5 | X |
|  |  |  |  |  |  |  |
| 11655 | EXTEM | CT | 5 | 8.88 | 5 | 4.85 |
|  |  | A5 | 5 | 2.85 | 5 | 3.82 |
|  |  | A10 | 5 | 2.48 | 5 | 3.39 |
|  |  | A20 | 5 | 2.27 | 5 | 3.04 |
|  |  | MCF | 5 | X | 5 | X |
|  | INTEM | CT | 5 | 9.23 | 5 | 2.54 |
|  |  | A5 | 5 | 1.30 | 5 | 2.45 |
|  |  | A10 | 5 | 1.34 | 5 | 2.16 |
|  |  | A20 | 5 | 1.10 | 5 | 0.00 |
|  |  | MCF | 5 | X | 5 | X |
|  | FIBTEM | A5 | 5 | 1.86 | 5 | 1.93 |
|  |  | A10 | 5 | 2.60 | 5 | 3.36 |
|  |  | A20 | 5 | 2.40 | 5 | 1.53 |
|  |  | MCF | 5 | X | 5 | X |
|  | HEPTEM | CT | 5 | 3.50 | 5 | 2.33 |
|  |  | A5 | 5 | 1.39 | 5 | 3.07 |
|  |  | A10 | 5 | 0.00 | 5 | 2.72 |
|  |  | A20 | 5 | 1.18 | 5 | 3.13 |
|  |  | MCF | 5 | X | 5 | X |
|  |  |  |  |  |  |  |
| 11657 | EXTEM | CT | 5 | 2.49 | 5 | 1.73 |
|  |  | A5 | 5 | 1.85 | 5 | 5.02 |
|  |  | A10 | 5 | 1.39 | 5 | 4.42 |
|  |  | A20 | 5 | 1.27 | 5 | 4.16 |
|  |  | MCF | 5 | X | 5 | X |
|  | INTEM | CT | 5 | 2.29 | 5 | 1.45 |
|  |  | A5 | 5 | 2.08 | 5 | 3.21 |
|  |  | A10 | 5 | 1.22 | 5 | 2.83 |
|  |  | A20 | 5 | 1.12 | 5 | 1.98 |
|  |  | MCF | 5 | X | 5 | X |
|  | FIBTEM | A5 | 5 | 3.88 | 5 | 4.80 |
|  |  | A10 | 5 | 3.73 | 5 | 4.25 |
|  |  | A20 | 5 | 3.41 | 5 | 3.99 |
|  |  | MCF | 5 | X | 5 | X |
|  | HEPTEM | CT | 5 | 0.69 | 5 | 1.27 |
|  |  | A5 | 5 | 2.60 | 5 | 3.07 |
|  |  | A10 | 5 | 3.30 | 5 | 2.07 |
|  |  | A20 | 5 | 2.39 | 5 | 2.91 |
|  |  | MCF | 5 | X | 5 | X |
|  |  |  |  |  |  |  |
| 11658 | EXTEM | CT | 5 | 12.89 | 5 | 4.96 |
|  |  | A5 | 5 | 2.85 | 5 | 11.35 |
|  |  | A10 | 5 | 2.66 | 5 | 10.03 |
|  |  | A20 | 5 | 2.44 | 5 | 8.84 |
|  |  | MCF | 5 | X | 5 | X |
|  | INTEM | CT | 5 | 3.40 | 5 | 1.91 |
|  |  | A5 | 5 | 1.29 | 5 | 8.70 |
|  |  | A10 | 5 | 1.46 | 5 | 7.46 |
|  |  | A20 | 5 | 1.36 | 5 | 6.68 |
|  |  | MCF | 5 | X | 5 | X |
|  | FIBTEM | A5 | 5 | 2.00 | 5 | 6.71 |
|  |  | A10 | 5 | 1.80 | 5 | 7.69 |
|  |  | A20 | 5 | 3.41 | 5 | 7.25 |
|  |  | MCF | 5 | X | 5 | X |
|  | HEPTEM | CT | 5 | 2.32 | 5 | 2.80 |
|  |  | A5 | 5 | 2.14 | 5 | 8.29 |
|  |  | A10 | 5 | 2.51 | 5 | 3.64 |
|  |  | A20 | 5 | 2.97 | 5 | 4.12 |
|  |  | MCF | 5 | X | 5 | X |

EXTEM: thromboelastometry with extrinsic activation, INTEM: thromboelastometry with intrinsic activation, FIBTEM: thromboelastometry with cytochalasin D-mediated platelet inhibition, HEPTEM: thromboelastometry with heparinase, ROTROL: ROTEM manufacturer QC materials, normal and abnormal levels

CT: clotting time in seconds, A5: amplitude at 5 minutes, A10: amplitude at 10 minutes, A20: amplitude at 20 minutes, MCF: maximum clot formation

X^1^ Due to errors in data handling, imprecision for each instrument could not be resolved for MCF. However, pooled results are available in **Table 2.**”

**Supplemental Table 2. Calculated Mahalanobis Distances for EXTEM, INTEM, FIBTEM and HEPTEM Assays**

| Sample | EXTEM | INTEM | FIBTEM | HEPTEM |
| --- | --- | --- | --- | --- |
| 1 | 3.67 | 1.77 | 6.65 | 6.87 |
| 2 | 3.78 | 0.83 | 7.21 | 3.49 |
| 3 | 1.18 | 1.15 | 7.38 | 0.87 |
| 4 | 4.07 | 5.78 | 6.78 | 8.77 |
| 5 | 4.04 | 0.59 | 2.03 | 3.50 |
| 6 | 3.63 | 2.35 | 1.16 | 2.14 |
| 7 | 6.02 | 0.39 | 4.14 | 3.33 |
| 8 | 3.60 | 4.36 | 2.83 | 1.56 |
| 9 | 4.03 | 1.67 | 2.88 | 3.36 |
| 10 | 6.18 | 7.27 | 1.16 | 5.09 |
| 11 | 10.30 | 1.63 | 1.74 | 5.20 |
| 12 | 6.37 | 5.17 | 1.18 | 7.62 |
| 13 | 2.28 | 1.67 | 3.82 | 3.82 |
| 14* | 5.41 | 2.23 | 9.36* | 10.03 |
| 15* | 19.94* | 5.62 | 4.07 | 5.82 |
| 16* | 13.73* | 5.31 | 11.92* | 7.06 |
| 17 | 3.38 | 5.64 | 3.70 | 3.46 |
| 18 | 7.27 | 4.97 | 1.99 | 3.73 |
| 19 | 11.75 | 3.02 | 1.74 | 3.66 |
| 20 | 4.93 | 1.79 | 4.27 | 1.23 |
| 21 | 1.44 | 3.94 | 5.08 | 5.90 |
| 22 | 6.90 | 1.33 | 1.35 | 10.32 |
| 23 | 4.11 | 1.54 | 2.51 | 8.17 |

* Sample identified as outlier, excluded from analysis after additional review and donor interview

CT: clotting time in seconds, A5: amplitude at 5 minutes, A10: amplitude at 10 minutes, A20: amplitude at 20 minutes, MCF: maximum clot formation

**Supplemental Table 3- Patient Demographics**

|  |  |  |  |  |  |
| --- | --- | --- | --- | --- | --- |
|  | **L&D^a^** | **Trauma** | **CVOR^b^** | **Liver Transplant** | **Total** |
| Number | 15 | 10 | 9 | 17 | 51 |
| Median Age (IQR) | 34 (30-38) | 44 (32-57) | 70 (67-73) | 62 (41-73) | 49 (34-70) |
| Male (%) | 0 (-%) | 7 (70%) | 8 (89%) | 10 (59%) | 25 (49%) |
| Self identify as White | 13 (87%) | 6 (60) | 9 (100%) | 17 (100%) | 45 (88%) |
| Lab Results |  |  |  |  |  |
| INR (0.90-1.20)^d^ | X^c^ | 1.2 (1.13-1.28) | 1.5 (1.2-1.65) | 1.6 (1.4-2.1) | 1.5 (1.2 -1.83) |
| PTT (28-38, s) | x | 29 (23-34.8) | 32 (29.2-36.5) | 37 (29.5-63.5) | 34 (28-37.5) |
| Fibrinogen (170-400, mg/dL) | x | 248 (180-316) | 230 (178-281) | 178 (155-255) | 193 (160-272) |
| Platelets (150-400, x10^3^ µL) | 207 (177-256) | 104 (58-179) | 111 (78-188) | 91 (57-172) | 113 (78-201) |

^a^Labor and Delivery, ^b^Cardiovascular OR, ^c^x = no results available for the population, ^d^all lab values are the (reference interval, units of measure).

**Supplemental Figure 1 Comparison between the ROTEM delta and sigma.** 71 patients were assessed on each instrument from multiple clinical locations. Healthy controls-20, L&D-15, CVOR-9, Liver transplant – 17, trauma- 10. The black line is the best fit regression line for all specimens and the gray dashed line is the line of identity.

**Supplemental Table 4 – Deming regression of the ROTEM delta and sigma between patient groups**
